# C3a‐C3aR Signaling Modulates Neutrophil Extracellular Traps Formation and Contributes to Blood–Brain Barrier Injury After Stroke

**DOI:** 10.1002/cns.71239

**Published:** 2026-11-09

**Authors:** Qingtong Zhang, Feng Xu, Chenyao Jian, Nian Li, Tianhao Zhang, Daling Deng, Mingda Liu, Manlin Duan

**Affiliations:** ^1^ Department of Anesthesiology Jinling Hospital, Affiliated Hospital of Medical School, Nanjing University Nanjing Jiangsu China; ^2^ Department of Anesthesiology The First Affiliated Hospital, Zhejiang University School of Medicine Hangzhou China; ^3^ Department of Anesthesiology Nanjing BenQ Medical Center, The Affiliated BenQ Hospital of Nanjing Medical University Nanjing Jiangsu China; ^4^ Department of Anesthesiology Union Hospital, Tongji Medical College, Huazhong University of Science and Technology Wuhan China; ^5^ The Core Laboratory Nanjing BenQ Medical Center, The Affiliated BenQ Hospital of Nanjing Medical University Nanjing Jiangsu China; ^6^ Department of Anesthesiology Nanjing Tianyinshan Hospital, The First Affiliated Hospital of China Pharmaceutical University Nanjing China

**Keywords:** complement, neuroinflammation, neutrophil

## Abstract

**Background:**

Stroke causes severe neurological damage, blood–brain barrier (BBB) dysfunction, complement activation, and neutrophil infiltration. How complement signaling interacts with infiltrating neutrophils in BBB dysfunction remains unclear.

**Purpose:**

This study aims to elucidate how the C3a‐C3aR axis modulates neutrophil extracellular traps (NETs) formation and thereby contributes to BBB disruption following stroke.

**Methods:**

A transient middle cerebral artery occlusion (tMCAO) rat model was used to induce ischemic stroke. Neurological function, infarct volume, and BBB integrity were assessed using behavioral tests, TTC staining, electron microscopy, and BBB permeability assays. Molecular and histological analyses, including RNA sequencing, immunostaining, HE staining, Nissl staining, ELISA, RT‐qPCR, Co‐IP, and western blotting, were performed to evaluate complement activation, NETs, and inflammatory responses. The C3a‐C3aR axis and NETs formation were pharmacologically modulated to determine their roles in post‐stroke BBB damage.

**Results:**

BBB damage, neutrophil infiltration, and neurobehavioral deficits are prominent after stroke. On the third day after the stroke, BBB damage is most pronounced. RNA sequencing revealed that inflammatory responses, complement activation, and NETs formation were identified as central mechanisms. Key driver analysis confirmed that C3a‐C3aR signaling was associated with neutrophil responses and NETs formation. Increased numbers of neutrophils infiltrated the brain parenchyma and formed NETs after stroke, in association with BBB damage and neurological injury. C3aR was expressed in neutrophils, consistent with a potential role of C3a‐C3aR signaling in neutrophil responses. Inhibiting NETs formation mitigated these poor functional outcomes, ameliorating BBB dysfunction. C3a‐C3aR signaling was associated with NETs formation and BBB disruption and neurological injury. Suppression of C3a‐C3aR signaling reduced NETs formation and was associated with attenuated BBB disruption and neurological impairment. Conversely, activation of C3a‐C3aR signaling was associated with increased NETs formation, BBB disruption, and neurological damage.

**Conclusion:**

Thus, our results indicate that C3a‐C3aR signaling is associated with NETs formation, BBB damage, and neurological impairment after stroke and may represent a potential target for further investigation.

## Graphical abstract

Following ischemic stroke, C3a‐C3aR signaling was associated with increased neutrophil extracellular traps (NETs) formation, blood–brain barrier (BBB) disruption, and neurological impairment. Pharmacological modulation of C3a‐C3aR signaling or NETs formation attenuated BBB injury and improved neurological outcomes, supporting a potential role of C3a‐C3aR‐NETs interactions in post‐stroke brain injury.

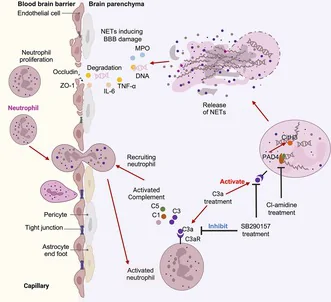

## 1 Introduction

Stroke is the second largest cause of death worldwide and the main source of lifelong disability, making it a major global health concern [1, 2]. Stroke is characterized by an abrupt interruption of blood supply to the brain, which can cause significant damage to the nervous system and impair functioning. The most prevalent type of stroke is an ischemic stroke (IS), which is caused by a brain artery blockage that sets off a series of pathological processes [3].

A serious outcome of ischemic stroke is the rupture of the blood–brain barrier (BBB), which is essential for preserving the homeostasis of the central nervous system. BBB dysfunction is a key link in ischemic stroke, characterized by the degradation of BBB structure proteins and elevated permeability [4, 5]. When cerebral ischemia occurs, the injured endothelial cells and surrounding astrocytes release a series of chemical mediators, which aggravate the destruction of the BBB [6, 7, 8]. Due to the change in the permeability of the damaged BBB, neutrophils in the peripheral blood move across the endothelium and enter the brain parenchyma [9], affecting the homeostasis of the brain, inducing neuroimmune responses [10], and interacting with central immune cells [11, 12], further leading to an imbalance in central homeostasis and forming a vicious cycle [13].

These neutrophils, which migrate from the peripheral circulation, can release neutrophil extracellular traps (NETs) [14], which are networks containing DNA, histones, and various proteins like cytotoxic granule‐derived proteins designed to trap and neutralize pathogens [15, 16]. NETs have several ways to induce BBB dysfunction including inflammatory cascade [17], structural injury [18], and thrombus formation [14, 18]. To sum up, during a stroke, NETs play a major role in disrupting the BBB, increasing inflammation, causing damage to neuronal cells, and making the prognosis worse.

C3 (Complement 3), as a central protein in the complement system, participates in the neuroinflammatory response. C3 is broken down into pieces upon activation, one of which is the strong inflammatory peptide C3a [19]. C3aR is the receptor for C3a [19], which is present on the surface of some immune cells and regulates some functions of cells [20, 21]. Currently, in stroke, there is limited evidence regarding the role of the C3a‐C3aR axis in NETs and BBB function. The relationship between C3a‐C3aR signaling, NETs formation, and BBB dysfunction remains unclear.

Considering that C3aR signaling has been connected to a number of inflammatory events, understanding how it affects NETs regulation and BBB breakdown may shed light on the pathophysiology of stroke. Determining how C3a‐C3aR signaling is associated with NETs formation and BBB injury following stroke, particularly in the acute setting examined here, was the goal of this work.

## 2 Methods

### 2.1 Animals

All experimental protocols were granted by the Institutional Animal Care and Use Committee (IACUC) of Nanjing Medical University (Nanjing, China) with approval number IACUC‐2404060. Male SD (Sprague–Dawley) rats (Weight: 200–250 g, approximately 6–8 weeks old) were sourced from GemPharmatech (Nanjing, China). There are four experiments involved in animal treatment in this study. First, we established the sham and the transient middle cerebral artery occlusion (tMCAO) models. Second, we divided tMCAO rats into 2 groups: those receiving SB290157 trifluoroacetate (C3aR antagonist) at 5 mg/kg and vehicle control. Third, we divided tMCAO rats into two groups: those receiving Cl‐amidine hydrochloride (a NETs inhibitor) and vehicle control. Fourth, we divided tMCAO rats into two groups: those receiving intranasal administration of C3a protein and vehicle control. All animal experiments were performed in the Animal Experiment Center of Nanjing BenQ Medical Center, The Affiliated BenQ Hospital of Nanjing Medical University or Jinling Hospital, Affiliated Hospital of Medical School, Nanjing University. Animals were euthanized by an intraperitoneal overdose of sodium pentobarbital (100 mg/kg), in accordance with institutional guidelines.

### 2.2 Cerebral Ischemia–Reperfusion Injury Model and Animal Grouping

Cerebral ischemia–reperfusion (I/R) injury was induced using the tMCAO method, as previously described in our work [22, 23]. All MCAO surgical instruments are strictly disinfected at high temperatures, and iodophor disinfection is carried out before and after tMCAO surgery. In brief, adult SD rats were anesthetized with sodium pentobarbital (40 mg/kg, intraperitoneally), and the right common carotid artery (CCA), vagus nerve, external carotid artery (ECA), and internal carotid artery (ICA) were exposed. A filament (RWD, Shenzhen, China) was inserted into the ICA to a depth of 18.5 ± 0.5 mm, initiating cerebral ischemia. After 60 min of tMCAO, the filament was removed, and reperfusion was allowed. During the surgery, the respiratory cycle was maintained stable, and the rectal temperature was monitored and kept at about 37°C. Neurological deficits were assessed using the Longa scoring system in all rats after tMCAO induction. The scoring criteria were as follows. There were four levels of neurological impairment: 0 indicates no deficits at all; 1 indicates a mild impairment; 2 indicates a moderate impairment; 3 indicates a severe impairment; and 4 indicates no spontaneous walking with reduced consciousness. Each level is represented by a different walking pattern. To be included in the study, rats with a Longa score of one to three were chosen.

### 2.3 Behavioral Assessment of Neurological Function

A modified neurological severity score (mNSS) was conducted after the stroke to assess global sensorimotor function as previously described [22]. The total score is 18 points, with higher scores indicating greater neurological impairment. A modified sticky‐tape test (MST) was conducted after stroke to assess somatosensory dysfunction [24]. Briefly, a non‐removable tape sleeve is wrapped around the animal's paw for the test, and the animal's reaction time to the stimulus removal is timed in 30 s. The time that the animal spent focusing on this stimulus was recorded and tested in both front paws. The ratio of interest is calculated by dividing the duration of the animal's attention to the stimuli in seconds by 30 s. The ratio of interest of the left front paw to the right front paw was the final result, with a higher ratio indicating better somatosensory function.

The open field test (OFT) was employed to assess neurobehavioral performance such as motor function, depressive‐like behavior, and exploratory activity after stroke [25]. The apparatus consisted of a square box (1 m × 1 m × 0.5 m). Behavioral parameters, including total distance and average speed, were recorded after stroke. The behavioral experimenter was blind to the grouping information. Duration of OFT was set to 5 min.

### 2.4 Survival Curve Analysis

Survival analysis was plotted to evaluate the influence of the C3a‐C3aR axis on post‐tMCAO prognosis. Survival curves were plotted during 7 days after inducing stroke models. Survival curves were generated by using the Kaplan–Meier method, with comparisons between the C3aRA group and the vehicle group. A significant log‐rank result was shown to confirm the effect of the C3a‐C3aR axis on survival conditions after stroke.

### 2.5 Measurement of Cerebral Infarct Size

Cerebral infarct size was quantified using TTC (Triphenyltetrazolium chloride) staining, as previously described [23]. After the stroke, rats were anesthetized with sodium pentobarbital, and brain tissues were collected. Coronal brain sections (thickness of 2 mm) were placed in TTC solution (2%) at 37°C for 18 min and immersed in 4% PFA (Paraformaldehyde). Ischemic areas were identified as white, while normal areas were stained red. Infarct volume was quantified using ImageJ software: Infarct volume (cm^3^) = total infarct volume (cm^2^) × 0.2 (cm).

### 2.6 Hematoxylin and Eosin (HE) and Nissl Staining

Nissl staining was conducted to estimate Nissl bodies and neuronal survival [22]. HE staining is employed to detect inflammatory cells. Paraffin‐embedded brain slices were deparaffinized, rehydrated, and stained following the manual of the HE stain kit (Solarbio, China, catalog number: G1120) and Nissl stain kit (Solarbio, China, catalog number: G1430). Images of HE and Nissl staining were scanned by a microscope with a bright field. The numbers of inflammatory cells and Nissl bodies were observed in the images.

### 2.7 RNA‐Seq After tMCAO

Cortical tissues of rats were collected 3 days after establishing animal models. Then, samples were sent to BGI (Beijing Genomics Institute, China) for further processing. The protocol for RNA‐seq was as described before [26]. In brief, total RNA was extracted. Afterward, RNA samples were denatured to unfold secondary structure and enriched by oligo (dT)‐attached magnetic beads. The mRNA was fragmented and converted to cDNA. After end‐repairing double‐stranded cDNA fragments, a single “A” nucleotide was added, followed by adaptor ligation. The program and PCR reaction apparatus were set up and configured to amplify their product, followed by circularization to produce single‐stranded circularized products. Libraries were sequenced on the DNBSEQ platform of BGI. Then, the sequencing data were filtered and stored in FASTQ format. Dr. Tom (https://biosys.bgi.com) from BGI, based on cloud bioinformatics systems, was used for the analysis and data mining. Differential expression analysis was conducted via DESeq2 with (*p* value < 0.05, fold change > 2). GO, KEGG, and GSEA (Gene set enrichment analysis) enrichment analyses of annotated differentially expressed genes (DEGs) were carried out to gain insight into the phenotypic changes (adjusted *p* value < 0.05). Key driver analysis was performed based on a network of interactions by Dr. Tom Multi‐omics Data Mining System.

### 2.8 Electron Microscopy

After perfusion with normal saline, the cortical tissues of rats were collected 3 days after establishing the animal models. Then, brain samples were sent to JAVIS (Wuhan, China) for further processing. Brain samples were immersed in 2.5% glutaraldehyde for 2 days and then washed with phosphate buffer three times. The cortex was immersed in 1% osmium tetroxide for 2 h, followed by washing with phosphate buffer three times. Tissues were dehydrated through a graded ethanol series (50%, 70%, 80%, 90%, and 100%) and embedded in epoxy resin, then cut into brain sections. The brain slices were stained with uranyl acetate for 15 min. Ultimately, the electron microscope was used to capture images of structure with BBB.

### 2.9 BBB Leakage Test

For BBB leakage assessment, Evans blue and Sulfo‐NHS‐Biotin were used to assess BBB permeability, which are typical methods to examine BBB permeability [27, 28]. For the Evans blue test, Evans blue dye (2%, Sigma‐Aldrich, USA, catalog number: E2129) was administered intravenously via the femoral vein at 4 mL/kg. Two hours later, rats were anesthetized and perfused with normal saline to remove intravascular dye. Brains were collected for quantification. The tissue samples were homogenized in trichloroacetic acid and centrifuged, and then the supernatant was diluted with ethanol. The absorbance of the samples was measured at 620 nm using a spectrophotometer. For the biotin assay, rats were injected with biotin (Thermo Fisher Scientific, USA, catalog number: 21217) at 5 mg/mL in PBS through femoral veins, and the dosage is 2 mL/kg. After 1 h, rats were anesthetized and perfused transcardially with normal saline. Brain tissues were gathered, fixed, and dehydrated. Brain sections were prepared by using a freezing microtome. Brain sections were incubated with Alexa Fluor 594 conjugates of streptavidin (Thermo Fisher Scientific, USA, catalog number: S11227) for 2 h at room temperature. Imaging was conducted by a fluorescence microscope to visualize BBB leakage.

### 2.10 ELISA (Enzyme‐Linked Immunosorbent Assay)

Cortex tissue and blood were collected 3 days after the tMCAO. The cortex tissue was homogenized in PBS, centrifuged, and the supernatant was gathered for ELISA. Blood from the external jugular vein was stored in a 1.5 mL tube at room temperature for 30 min, then centrifuged, and serum was gathered for subsequent ELISA. The levels of complement components and cytokines were measured by ELISA. Rat C3a ELISA kit (Bioswamp, Wuhan, China, catalog number: RA20070), Rat interleukin‐6 ELISA Kit (Bioswamp, Wuhan, China, catalog number: RA20607), Rat TNF‐α ELISA kit (RUIXIN BIOTECH, Quanzhou, China, catalog number: RX302058R), Rat IFN‐β ELISA kit (RUIXIN BIOTECH, Quanzhou, China, catalog number: RX302909R), and Rat cfDNA ELISA kit (Mybiosource, USA, catalog number: MBS7273611) are used in this paper. Protocols for ELISA in this study follow the manuals of these kits. OD values are measured by a spectrophotometer. Then, a standard curve was fitted to measure the concentrations of the samples.

### 2.11 Routine Hematology Tests in Rats

Blood from the external jugular vein was collected 3 days after the tMCAO. Blood samples were stored in purple EDTA (ethylenediaminetetraacetic acid) tubes. Then, the tubes were sent to Servicebio (Wuhan, China) for a five‐part differential analysis of routine blood (animal peripheral blood). The test involves using a hematology analyzer to calculate the percentage and count of five types of white blood cells (neutrophils, lymphocytes, monocytes, eosinophils, and basophils) in the peripheral blood of animals.

### 2.12 C3a‐C3aR Axis Antagonizing Treatment

SB290157 trifluoroacetate (MCE, USA, catalog number: HY‐101502A) has been reported as a C3aR antagonist and was used here to inhibit C3a‐C3aR signaling [29]. We set the dosage at 5 mg/kg. Following the MCE manual (https://www.medchemexpress.com/SB290157_trifluoroacetate.html), SB290157 powder was dissolved in working solution with 10% DMSO (dimethyl sulfoxide) + 40% PEG300 (polyethylene glycol 300) + 5% Tween‐80 + 45% normal saline, and SB290157 working solution was set at 0.5 mg/mL. The working solution without SB290157 was set as vehicle control. SB290157 was administered intraperitoneally for 5 consecutive days as a pretreatment starting 2 days before tMCAO.

### 2.13 Intranasal Treatment of C3a Protein

C3a is an anaphylatoxin generated during complement activation. Intranasal treatment of C3a is reported to be effective in elevating the level of C3a in the brain [30]. The purified human C3a (MCE, USA, catalog number: HY‐P7862) was dissolved in PBS (Phosphate‐buffered saline) at 10 μg/mL. The dose for intranasal treatment was set at 1 μg/kg. Using a micropipette, 20 μL of the C3a protein solution was administered intranasally. Each nostril received an equal portion of the total volume (10 μL per nostril). This procedure was performed daily for 7 consecutive days, beginning 4 days prior to tMCAO.

### 2.14 NETs Inhibition

Cl‐amidine hydrochloride (MCE, USA, catalog number: HY‐100574A) was proven to be effective in inhibiting NETs formation, which is a pan‐PAD inhibitor with activity against PAD4 [9, 31]. The dosage of Cl‐amidine hydrochloride was set at 10 mg/kg. Following the MCE manual, Cl‐amidine hydrochloride was dissolved in PBS. The Cl‐amidine hydrochloride working solution was set at 0.5 mg/mL. The working solution without Cl‐amidine hydrochloride was set as the vehicle control. Cl‐amidine hydrochloride was administered intraperitoneally immediately after tMCAO and once daily for 3 consecutive days.

### 2.15 Immunofluorescence Staining and Immunohistochemistry

Immunohistochemistry (IHC) and immunofluorescence staining (IF) were used to localize and quantify proteins in the cortex. IF was performed using frozen sections. After blocking with 0.3% Triton X‐100 and 5% BSA (Bovine serum albumin), slices were added with primary antibodies overnight at 4°C. Following PBS washes, the sections were incubated with secondary antibodies for 1 h at room temperature. The primary antibodies included HIS48 (1/200, abcam, UK, catalog number: ab33760), CitH3 (1/500, abcam, UK, catalog number: ab214810), C3aR (1/200, Santa Cruz, USA, catalog number: sc‐133172), MPO (1/200, Proteintech, China, catalog number: CL594‐66177), CD31 (1/200, R&D, USA, catalog number: AF3628), Occludin (1/100, Proteintech, China, catalog number: 27260‐1‐AP), and ZO‐1 (1/100, Proteintech, China, catalog number: 21773‐1‐AP).

IHC utilized diaminobenzidine (DAB) for chromogenic detection. Briefly, brain slices were placed in a microwave container with an antigen retrieval buffer for heating and maintaining heat for 10 min following cooling. The brain slices were incubated in 3% hydrogen peroxide to block endogenous enzyme activity. After washing, brain sections were incubated with 5% BSA to block non‐specific binding sites. The brain slices were incubated with PAD4 antibody (1/200, abcam, UK, catalog number: ab5103) overnight. After washing, the brain slices were incubated with secondary antibodies with HRP‐DAB. For chromogenic detection, streptavidin‐HRP was added to brain slices for 20 min following the addition of the DAB solution. Brain slices were rinsed in water to stop the reaction following mounting. Positive cells in IF and IHC were quantified to assess the staining of targets.

### 2.16 Quantitative Real‐Time Polymerase Chain Reaction (RT‐qPCR)

RT‐qPCR was utilized to measure the levels of complement and cytokine mRNA expression in the cortex. Total RNA was extracted using the RNAeasy Animal Long RNA Isolation Kit with Spin Column (Beyotime, China, catalog number: R0026) and reverse‐transcribed to cDNA using the HiScript II Q RT SuperMix for qPCR (Vazyme, China, catalog number: R223‐01). ChamQ Universal SYBR qPCR Master Mix (Vazyme, China, catalog number: Q711‐02) was used for quantitative analysis of genes. PCR is conducted according to the kit manuals. The primers for genes are listed in Table S1.

### 2.17 Western Blotting

As described in previous work, western blotting was conducted [22]. Briefly, proteins were extracted from the right cortex using lysis buffer, separated by SDS‐PAGE gel, and transferred to membranes. Membranes were immersed with 5% skim milk, incubated overnight with primary antibodies at 4°C, washed, and added with secondary antibodies for 2 h at room temperature. Enhanced chemiluminescence (ECL) reagents were used to detect bands of proteins using a chemiluminescence imaging system. We analyzed and quantified band intensity by ImageJ. Primary antibodies include C3 (1/500, Santa Cruz, USA, catalog number: sc‐28294), C3aR (1/500, Santa Cruz, USA, catalog number: sc‐133172), C1q (1/500, Santa Cruz, USA, catalog number: sc‐58920), ZO‐1 (1/500, Proteintech, China, catalog number:21773‐1‐AP), Occludin (1/500, Proteintech, China, catalog number: 27260‐1‐AP), PAD4 (1/1000, abcam, UK, catalog number: ab5103), CitH3 (1/1000, abcam, UK, catalog number: ab214810), H3 (1/1000, Proteintech, China, catalog number:17168‐1‐AP), GAPDH (1/2000, Abbkine, China, catalog number: KTD101), and β‐actin (1/2000, Abbkine, China, catalog number: KTD101). Enhanced chemiluminescence was used to visualize the proteins, and band intensity was quantified using ImageJ software.

### 2.18 Co‐IP (Co‐Immunoprecipitation)

To assess the association between C3‐derived material and C3aR in tissue lysates, Co‐IP was performed. Firstly, cortex lysate was extracted using a lysis buffer. C3aR antibody (Santa Cruz, USA, catalog number: sc‐133172) or anti‐rat IgG antibody (KTI1010‐CN, Abbkine, China) was incubated with magnetic beads in a mixer for 1 h at room temperature, following washing 3 times. Afterward, the lysate was added to the antibody‐beads complex and incubated overnight at 4°C with gentle rotation. The next day, the beads were washed 5 times to remove nonspecific binding. SDS sample buffer was used to elute protein complexes, following boiling the samples and preparing for SDS‐PAGE. Samples were loaded onto the SDS‐PAGE gel and separated proteins by electrophoresis, following the transfer of proteins to membranes and incubation of antibodies of C3 and C3aR. C3‐derived material and C3aR were detected to assess their association in tissue lysates by western blot.

### 2.19 Statistical Analysis

Data are presented as the mean ± standard error (SEM). Data were analyzed using one‐way or two‐way ANOVA followed by Tukey's multiple comparison tests for detecting the main effects of time and group and analyzing differences between groups at each time point. A two‐tailed Student's *t*‐test was employed for comparison between two groups. Survival curves were analyzed using Kaplan–Meier estimation, with comparisons made via the log‐rank and Gehan‐Breslow‐Wilcoxon tests. Statistical significance was set at *p* < 0.05. Data processing and analysis were performed using SPSS 22 and GraphPad Prism 9.

## 3 Results

### 3.1 The Neurobiological Impairment, BBB Leakage, and BBB Structural Disruption After tMCAO

In this study, the tMCAO model was established to examine stroke. Neurobehavioral tests, including mNSS score, MST, and OFT, were performed the day before modeling and 1 day, 3 days, 7 days, and 14 days after modeling (Figure 1A). In the mNSS scoring, the stroke group scored higher than the sham group from Day 1 to Day 14 following tMCAO (Figure 1B). In the MST experiment, the stroke group exhibited a lower MST ratio than the sham group from Day 1 to Day 14 following tMCAO (Figure 1C). OFT used in measuring the motor function showed that the stroke group had lower average speed and total travel distance than the sham group from Day 1 to Day 14 following tMCAO (Figure 1D–F). Figure 1E shows the trajectory plots and open field movement heatmap, which illustrates the distributions and movement of animals' activity. The red color indicates a higher activity frequency. From plots and heatmap, the stroke group showed reduced movement, particularly from Day 1 to Day 7 following tMCAO. Thus, after stroke, animals displayed impaired neurobehavioral performance, motor dysfunction, and sensory deficits.

**FIGURE 1:**
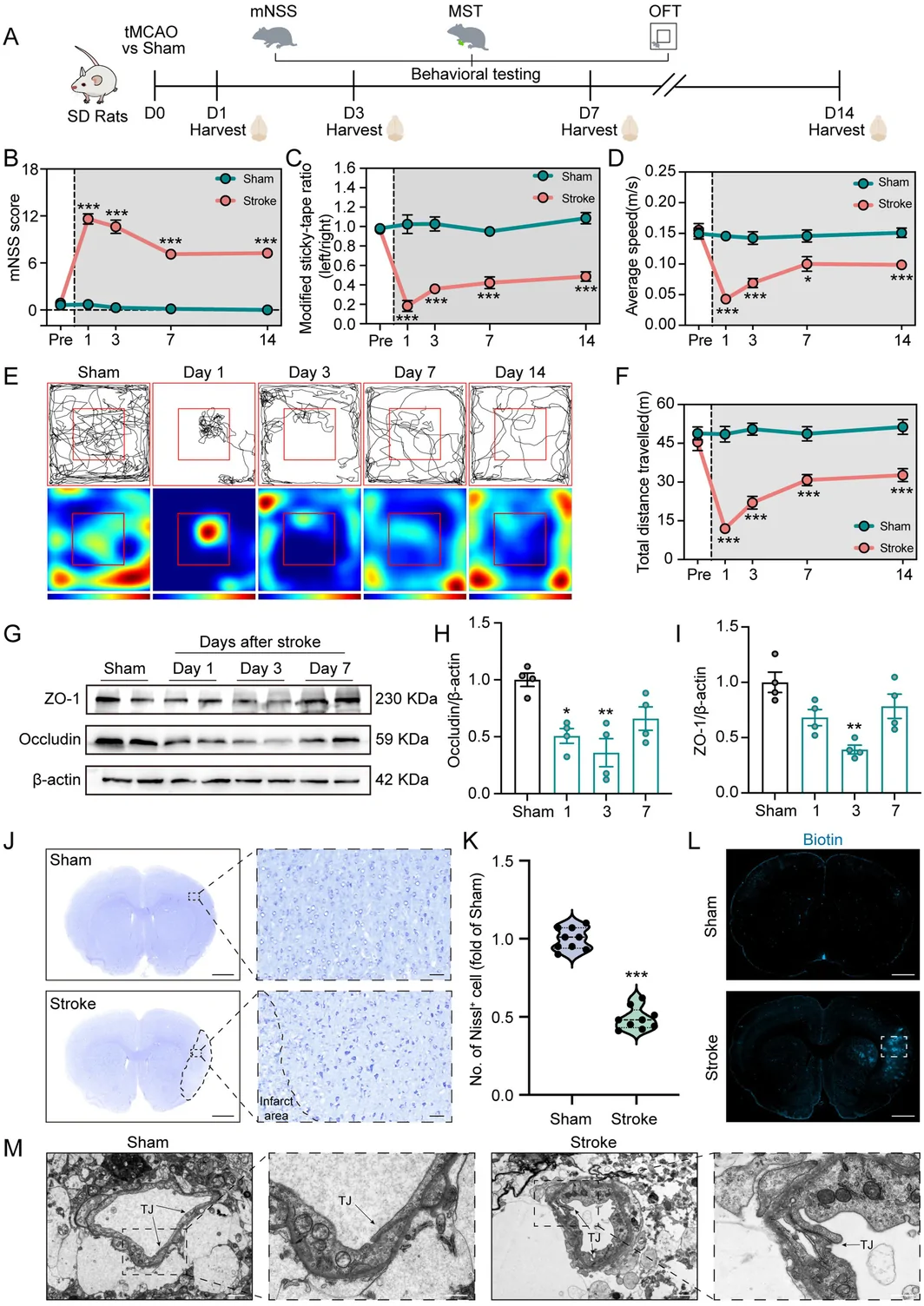
Neurobiological impairment, BBB leakage, and damage to BBB structure occurred after the stroke. (A) The flow diagram of an experiment in this part. tMCAO was used to induce stroke in an animal model. On Days 1, 3, 7, and 14 after tMCAO, neurobehavioral tests, including mNSS, MST, and OFT, were performed, and brain tissues were harvested. (B) mNSS score of rats on Days 1, 3, 7, and 14 after tMCAO (*n* = 8). (C) MST ratio of rats on Days 1, 3, 7, and 14 after tMCAO (*n* = 8). (D) Average speed of rats in OFT on Days 1, 3, 7, and 14 after tMCAO (*n* = 8). (E) Trajectory plots and open field movement heatmap of rats in OFT on Days 1, 3, 7, and 14 after tMCAO. (F) Total distance of rats in OFT on days 1, 3, 7, and 14 after tMCAO (*n* = 8). (G) WB bands of different groups. (H, I) Protein expression of ZO‐1 and Occludin normalized to β‐actin (*n* = 4). (J) Nissl staining of brain slices of sham and stroke group 3 days after tMCAO. (K) Quantitative analysis of Nissl bodies in the brain (*n* = 9 fields of view [FOVs] of 3 rats, 3 FOVs per rat). Scale bar = 2 mm or 50 μm (zoom). (L) BBB leakage test using biotin staining at 3 days after stroke. Scale bar = 1 mm. (M) Ultrastructure alteration of the tight junction near the endothelial cell at 3 days after stroke. Scale bar = 1 μm or 0.5 μm (zoom). Stroke group versus sham group, data as mean ± SEM. *p < 0.05, **p < 0.01, and ***p < 0.001.

These behavioral deficits were associated with neuronal injury, as shown in Figure 1J that Nissl bodies were reduced after stroke, indicating neuronal injury and loss. These neurobehavioral changes, indicating a deficit in motor and sensory impairment, may be closely correlated with BBB disruption, such as BBB leakage and BBB structure breakdown leading to brain damage [32, 33]. BBB leakage and structure proteins were examined after the stroke. For the BBB structure disruption test, tight junction proteins as ingredients of BBB, like Occludin and ZO‐1, were detected after the stroke. As expected, the expression of ZO‐1 and Occludin decreased from Day 1 to Day 7 following tMCAO (Figure 1G–I). Especially on the third day after tMCAO, the expression levels of ZO‐1 and Occludin were notably downregulated. For better observing ultrastructural change on the third day after tMCAO, transmission electron microscopy was used in this study. After 3 days of stroke, higher levels of biotin were observed in the parenchyma of the striatum and cortex (Figure 1L). Moreover, as shown in Figure 1M, in the normal BBB structure of a sham group, continuous electron‐dense bands are located at the apical side of endothelial cells. However, in the stroke, there were evident features of BBB structure disruptions like discontinuous or dispersed tight junctions between endothelial cells, demonstrating BBB integrity loss. Biotin is commonly used as a tracer to estimate the BBB integrity and leakage. Thus, after the stroke, BBB leakage and BBB structure disruption occurred, leading to the loss of BBB integrity and an increase in BBB permeability.

### 3.2 RNA‐Seq Analysis Uncovers Inflammatory Response, Complement Cascades, NETs Formation, and Neutrophil Chemotaxis as Key Mechanisms After BBB Damage

To better elucidate the key mechanisms involved in BBB disruption, cortical tissues were collected for RNA‐seq on the third day after modeling when significant changes in BBB damage were observed. We identified 443 upregulated DEGs and 31 downregulated DEGs (Figure S1A,B). Then, those DEGs were included in the KEGG, GO, and Reactome enrichment analysis. We discovered that enrichment pathways or biological processes were related to the immune system process, innate immune response, inflammatory response, positive regulation of tumor necrosis factor α (TNF‐α) production, positive regulation of interleukin‐6 production (IL‐6), phagosome, complement and coagulation cascades, NETs formation, neutrophil degranulation, activation of C3 and C5, and so on (Figure S1C–E). To identify certain pathways and biological categories enriched in the DEGs, we performed the GSEA to reveal whether some pathways listed before, such as NETs formation, are activated or inhibited after BBB damage. Neutrophil chemotaxis (NES [normalized enrichment score] = 1.82, *p* value < 0.001), NETs formation (NES = 1.92, *p* value < 0.001), innate immune response (NES = 2.12, *p* value < 0.001), Complement and coagulation cascades (NES = 2.52, *p* value < 0.001), and inflammatory response (NES = 2.11, *p* value < 0.001) indicated strong positive enrichment (Figure S1F–J), potentially influencing BBB disruption after stroke. To confirm neutrophil migration into the brain parenchyma, HE staining was conducted, which exhibited that ischemic regions emerged with numerous infiltrated neutrophils' segmented nuclei (Figure S1K). We conducted a confirmatory test of the inflammatory response using ELISA, which revealed increased levels of TNF‐α and IL‐6, alongside decreased levels of IFN‐β in the brain (Figure S1L). Therefore, the inflammatory response, complement cascades, NETs formation, and their interactions emerge as key mechanisms following BBB damage on the third day after tMCAO.

### 3.3 After Stroke, Neutrophils Increased in Peripheral Blood, Infiltrated Into the Brain Parenchyma, and Formed NETs

At first, we identified that there was a marked increase of WBC (White blood cell) number and neutrophil number, alongside an increased neutrophil‐to‐lymphocyte ratio using animal routine hematology tests (Figure 2D–F). To confirm the neutrophil migration into the brain, we used lectin to label the endothelial cells and HIS48 (a marker of rat neutrophils) to stain the neutrophils. After stroke, there were numerous neutrophils infiltrating the brain parenchyma from the cerebral vasculature (Figure 2A–C), which exhibited neutrophils migrating from brain vessels (Figure 2A). MPO (Myeloperoxidase) and CitH3 (Citrullinated histone), released by neutrophils during the process of NETs formation, were used as markers of NETs formation. We observed numerous NETs formation in the brain parenchyma after stroke (Figure 2G,J). PAD4, by catalyzing the citrullination of histones and facilitating chromatin decondensation, is a crucial enzyme in regulating the formation of NETs. We detected that PAD4‐positive cells were increased after stroke (Figure 2H,I). Moreover, the expression of PAD4 and CitH3 was upregulated after stroke (Figure 2K–M). Hence, after stroke, neutrophil numbers increased in peripheral blood, and infiltration into the brain was accompanied by NETs formation and upregulation of PAD4.

**FIGURE 2:**
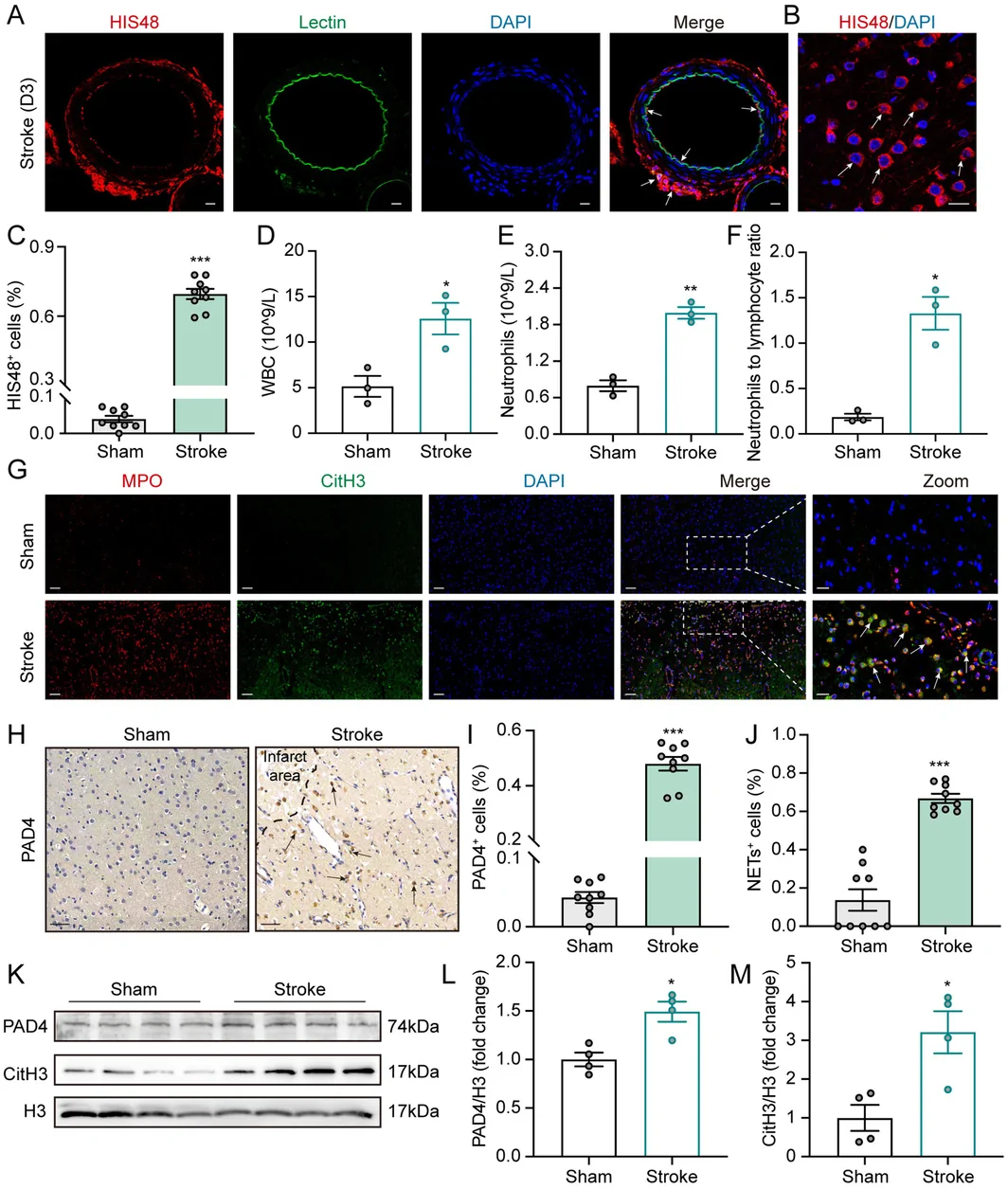
Neutrophils increased in peripheral blood and migrated into the brain, contributing to NETs formation. (A, B) Images of brain slices 3 days after stroke in the cortex, stained with antibodies against HIS48 (a marker of rats' neutrophils) and lectin for labeling vessels. Scale bar = 20 μm. (C) Quantification of HIS48‐positive cells (*n* = 9 FOVs of 3 rats, 3 FOVs per rat). (D–F) Quantification of WBC, neutrophils, and neutrophil‐to‐lymphocyte ratio 3 days after stroke (*n* = 3). (G) Images of NETs in sham and stroke groups 3 days after stroke. Scale bar = 50 μm or 20 μm (zoom). Arrows indicate the NETs in the stroke group. (H) Images of PAD4‐positive cells using IHC. Scale bar = 20 μm. Arrows indicate the PAD4‐positive cells. (I) Quantification of PAD4‐positive cells in both groups (*n* = 9 FOVs of 3 rats, 3 FOVs per rat). (J) Quantification of NETs in both groups (*n* = 9 FOVs of 3 rats, 3 FOVs per rat). (K) WB bands of different groups. (L, M) Protein expression of PAD4 and CitH3 normalized to H3 (*n* = 4). Stroke group versus sham group, data as mean ± SEM. *p < 0.05, **p < 0.01, and ***p < 0.001.

### 3.4 Inhibition of NET Formation Was Associated With Improved Neurobehavioral Outcomes, Reduced Infarct Volume, and Attenuated BBB Damage After Stroke

We reasoned that if NETs contributed to BBB disruption and its related neurobehavioral impairment, inhibiting NETs should improve BBB damage and neurobehavioral performance. To test this, we used Cl‐amidine, a pan‐PAD inhibitor with activity against PAD4 to suppress NETs formation. After inducing tMCAO, the animal models were injected with Cl‐amidine or vehicle for three continuous days (Figure 3A). Cl‐amidine treatment effectively reduced PAD4‐positive cell number and reduced the expression of PAD4 and CitH3, as well as NETs number (Figure 3D,H,K–O), suggesting a strong inhibitory effect of Cl‐amidine on NETs formation. Then, we tested its effects on neurobehavioral performance, infarct size, and BBB disruption. After inhibiting NETs formation, mNSS score, MST, and OFT displayed significant improvement after stroke, indicating sensory and motor function were rescued (Figure 3B,C,E–G). Furthermore, TTC staining also presented reduced infarct volume after Cl‐amidine treatment (Figure 3I,J). For BBB damage test, expression levels of ZO‐1 and Occludin were increased after NETs formation suppression, accompanied by reduced BBB leakage (Figure 3L,P–R). To further confirm the protective effect of NETs formation inhibition on BBB structure, we co‐labeled endothelial cells with CD31 and tight junctions with ZO‐1 or Occludin, observing that ZO‐1 and Occludin density near endothelial cells increased after stroke following Cl‐amidine treatment (Figure 3S–U). Our results implied that inhibition of NETs formation was associated with improved BBB integrity and neurological outcomes.

**FIGURE 3:**
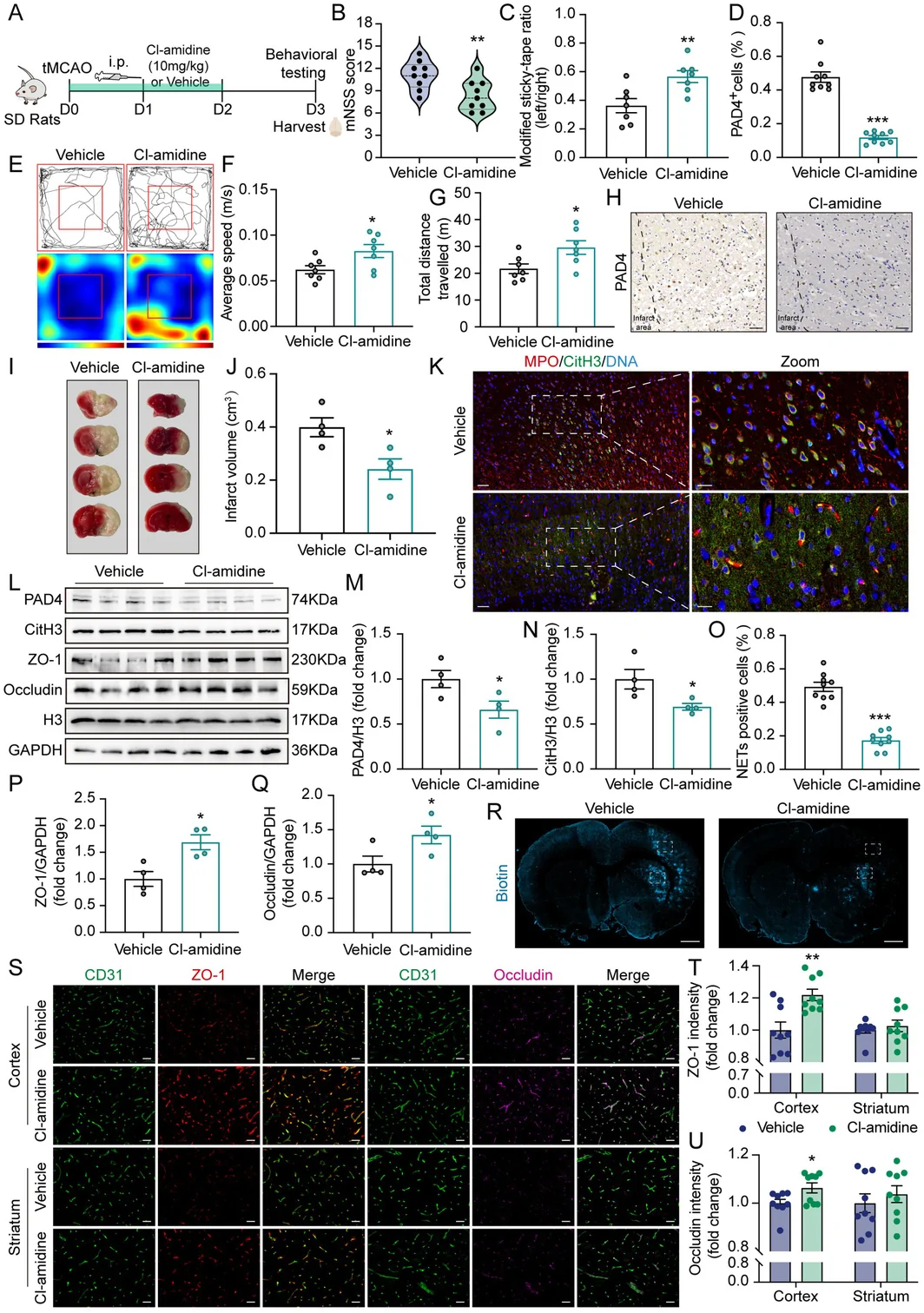
Inhibition of NETs formation improved neurobehavioral impairment, infarct size, and BBB damage after stroke. (A) The flow diagram of an experiment in this part. After tMCAO, the animals received intraperitoneal injection of inhibitor of NETs formation (Cl‐amidine) or vehicle for three continuous days. On day 3 after tMCAO, behavioral tests were performed, and brain tissue was harvested. (B, C) mNSS score (*n* = 9) and MST ratio (*n* = 7) in the vehicle and Cl‐amidine groups after suppressing NETs formation. (D) Quantification of PAD4‐positive cells in the vehicle and Cl‐amidine groups (*n* = 9 FOVs of 3 rats, 3 FOVs per rat). (E) Trajectory plots and open field movement heatmap in the vehicle and Cl‐amidine groups. (F, G) Average speed and total distance in the vehicle and Cl‐amidine groups (*n* = 7). (H) Images of PAD4‐positive cells using IHC, Scale bar = 20 μm. (I) TTC staining in the vehicle and Cl‐amidine groups. (J) Infarct volume in TTC staining (*n* = 4). (K) Images of NETs in the vehicle and Cl‐amidine groups. Scale bar = 50 μm or 20 μm (zoom). (L) WB bands of the vehicle and Cl‐amidine groups. (M‐N) Protein expression of PAD4 and CitH3 normalized to H3 (*n* = 4). (O) Quantification of NETs in the vehicle and Cl‐amidine groups (*n* = 9 FOVs of 3 rats, 3 FOVs per rat). (P, Q) Protein expression ZO‐1 and Occludin normalized to GAPDH (*n* = 4). (R) BBB leakage test using biotin staining. Scale bar = 1 mm. (S) Images of CD31 co‐labeled with ZO‐1 or Occludin in the vehicle and Cl‐amidine groups. Scale bar = 50 μm. (T, U) Quantification of ZO‐1 and Occludin density (*n* = 9 FOVs of 3 rats, 3 FOVs per rat). Cl‐amidine group versus vehicle group, data as mean ± SEM. *p < 0.05, **p < 0.01, and ***p < 0.001.

### 3.5 C3a‐C3aR Signaling Is Associated With NETs Formation

Gene sets related to complement and coagulation cascades and NETs formation are displayed in Figure 4A,C, such as C1qa, C3, C3aR, C4, and C5. Gene sets enriched in NETs formation and complement and coagulation cascades were all upregulated and covered C3 and C3aR. Additionally, we also performed KDA (Key driver analysis) to identify the crucial regulatory genes that influence the expression of other genes, which helped to pinpoint key genes driving biological processes and cellular behaviors. We discriminated the 10 key driver genes in Figure 4B, including C3aR. These data convince us that C3a‐C3aR signaling was associated with NETs formation and was identified as a potential regulatory node. Then we performed some confirmatory tests. We detected changes in complement proteins in transcription and protein levels. C1qa, C3, C3aR, C4, and C5 were upregulated in mRNA level after stroke, similar to RNA‐seq results (Figure 4C,D). C1q, C3, and C3aR were also elevated in protein levels after stroke (Figure 4E–H). IF revealed C3aR expression on neutrophils (Figure 4I,J), supporting their potential responsiveness to C3a‐C3aR signaling. Plus, Co‐IP results showed an association between C3‐derived material and C3aR in tissue lysates (Figure 4K); however, this assay does not establish direct ligand–receptor binding or identify whether intact C3 or a C3‐derived fragment accounts for the detected association. Our results support an association between C3a‐C3aR signaling, NETs formation, and BBB disruption after stroke.

**FIGURE 4:**
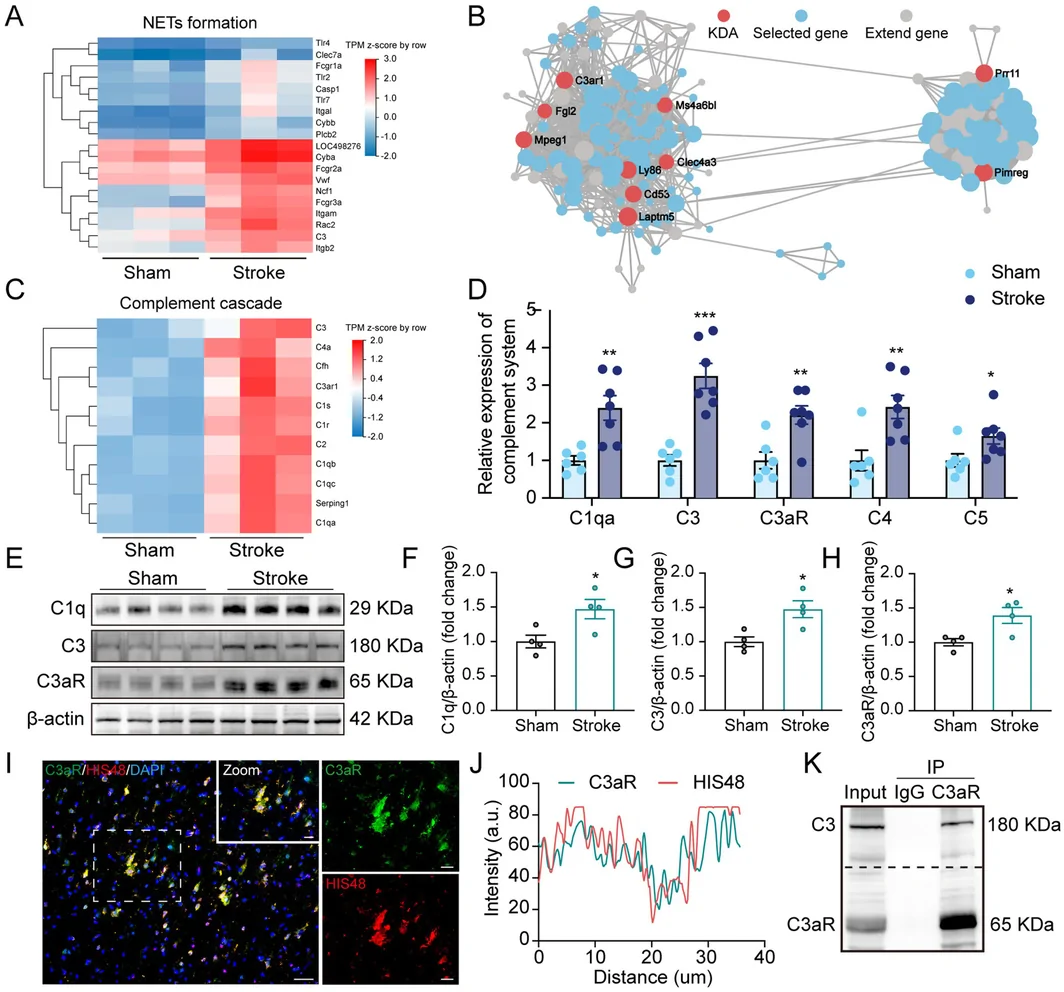
C3a‐C3aR signaling was identified as a potential key regulator associated with NETs formation. (A, C) Heatmap of genes enriched regarding the NETs formation and complement cascade (*n* = 3). C3 and C3aR are involved in complement cascade and NETs formation. (B) KDA provides that C3aR is a key driver in influencing the expression of other genes. (D) Quantification of mRNA of C1qa, C3, C3aR, C4, and C5 using RT‐qPCR 3 days after stroke (*n* = 6 in the sham group, and *n* = 7 in the stroke group). (E) WB bands in 2 groups 3 days after stroke. (F–H) Protein expression of C1q, C3, and C3aR normalized to β‐actin (*n* = 4). (I) C3aR expressed on the neutrophils. Scale bar = 50 μm or 20 μm (zoom). (J) The colocalization experiment showing the fluorescent signals of C3aR and HIS48. The density plot illustrates the fluorescence intensity in colocalized regions, indicating a spatial overlap. (K) Co‐IP results of C3aR and C3, showing an association between C3‐derived material and C3aR in tissue lysates. Stroke group versus sham group, data as mean ± SEM. *p < 0.05, **p < 0.01, and ***p < 0.001.

### 3.6 Inhibition of C3a‐C3aR Signaling Was Associated With Reduced NETs Formation, BBB Damage, and Neurological Impairment After Stroke

To examine the association of C3a‐C3aR signaling with NETs formation, we administered SB290157 for five consecutive days with treatment initiated before tMCAO (Figure 5A). This inhibition of C3a‐C3aR signaling was associated with reduced C3aR expression (Figure 5L,M). After C3a‐C3aR axis inhibition, we observed a significant decline in NETs formation, following specific manifestations: decreased NETs number, lowered cfDNA (circulating free DNA), and reduced CitH3 expression (Figure 5I,K,L,N). We further observed an association between C3a‐C3aR signaling inhibition and improved BBB integrity. When inhibiting the C3a‐C3aR axis and a subsequent reduction in NETs formation, BBB leakage and BBB structure were assessed. Evans blue dye was visually evident in the brain leakage area (Figure 5Q). We detected that by inhibiting the C3a‐C3aR axis, Evans blue dye leakage was decreased (Figure 5T). Moreover, biotin density near lectin‐labeled vessels also declined (Figure 5R,U). ZO‐1 and Occludin colocalized with CD31 were increased (Figure 5S,V,W). BBB ultrastructure damage was improved (Figure 5X). At last, after improving pathological impairment, we still examined the corresponding neurobehavioral changes and survival probability. As expected, the mNSS score, MST, and OFT all implied the sensory and motor functions were rescued, as well as increased survival probability after blocking the C3a‐C3aR axis (Figure 5B–H). Hence, our results indicate that inhibition of C3a‐C3aR signaling was associated with reduced NETs formation, BBB disruption, and neurological impairment in the peri‐ischemic experimental paradigm examined here.

**FIGURE 5:**
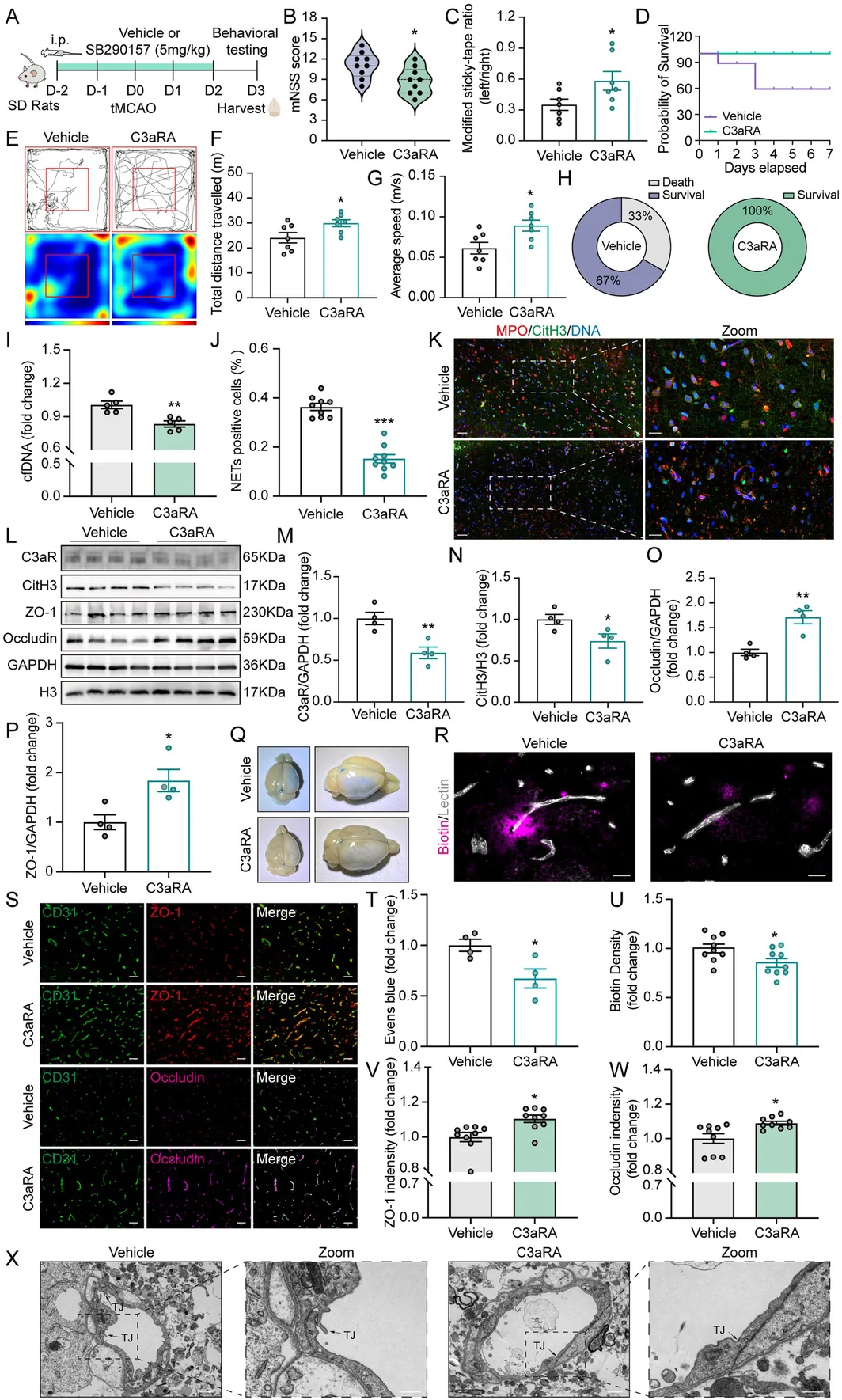
Inhibition of C3a‐C3aR signaling was associated with reduced NETs formation, BBB damage, and neurological impairment after stroke. (A) The flow diagram of an experiment in this part. The animals received intraperitoneal injections of the C3aR antagonist (C3aRA) SB290157 or vehicle for five consecutive days, starting 2 days before tMCAO. On day 3 after tMCAO, behavioral tests were performed, and brain tissue was harvested. (B, C) mNSS score (*n* = 9) and MST ratio (*n* = 7) in the vehicle and C3aRA groups. (D) Survival curve of the vehicle and C3aRA groups from day 0 to day 7 after stroke (*n* = 9). (E) Trajectory plots and open field movement heatmap in the vehicle and C3aRA groups. (F, G) Average speed and total distance in the vehicle and C3aRA groups (*n* = 7). (H) Survival rate in the vehicle and C3aRA groups (*n* = 9). (I) Quantification of cfDNA in the blood of the vehicle and C3aRA groups (*n* = 5). (J) Quantification of NETs in the vehicle and C3aRA groups (*n* = 9 FOVs of 3 rats, 3 FOVs per rat). (K) Images of NETs in the vehicle and C3aRA groups. Scale bar = 50 μm or 20 μm (zoom). (L) WB bands of the vehicle and C3aRA groups. (M–P) Protein expression of C3aR, CitH3, Occludin, and ZO‐1 normalized to GAPDH or H3 (*n* = 4). (Q) Image of BBB leakage using Evans blue dye in the vehicle and C3aRA groups. (R) Image of BBB leakage using biotin staining in the vehicle and C3aRA groups. Scale bar = 20 μm. (S) Images of CD31 co‐labeled with ZO‐1 or Occludin in the vehicle and C3aRA groups. Scale bar = 50 μm. (T, U) Quantification of Evans blue dye (*n* = 4) and biotin staining (*n* = 9 FOVs of 3 rats, 3 FOVs per rat) in the vehicle and C3aRA groups. (V, W) Quantification of CD31 co‐labeled with ZO‐1 or Occludin in the vehicle and C3aRA groups (*n* = 9 FOVs of 3 rats, 3 FOVs per rat). (X) Ultrastructure alteration of the tight junction near the endothelial cell. Scale bar = 1 μm and 0.5 μm. C3aRA group versus vehicle group, data as mean ± SEM. *p < 0.05, **p < 0.01, and ***p < 0.001.

### 3.7 Activating C3a‐C3aR Signaling Was Associated With Increased NETs Formation, BBB Damage, and Neurological Impairment After Stroke

Building upon the association observed after C3a‐C3aR inhibition, we next examined the effects of C3a administration on NETs formation, BBB disruption, and neurological injury. We used intranasal administration of C3a for 7 consecutive days, with treatment initiated before tMCAO (Figure 6A). C3aR and C3a were significantly upregulated after C3a treatment (Figure 6H,I,L). Then, we detected some indicators of NETs formation. We found that C3a administration was associated with increased NETs formation alongside increased expression of PAD4 and CitH3 (Figure 6G,J–M). For BBB disruption, C3a administration was associated with increased Evans blue dye and biotin leakage into the brain parenchyma (Figure 6P–S). Meanwhile, ZO‐1 and Occludin density were lowered, and BBB ultrastructure injuries were aggravated (Figure 6T–W), as well as decreased ZO‐1 and Occludin expression (Figure 6L,N,O). In terms of neurobehavioral tests, mNSS score, MST, and OFT all suggested that the sensory and motor dysfunctions were worsening (Figure 6B–F). These findings further support an association between C3a‐C3aR signaling, NETs formation, BBB damage, and neurological injury in the experimental paradigm examined here.

**FIGURE 6:**
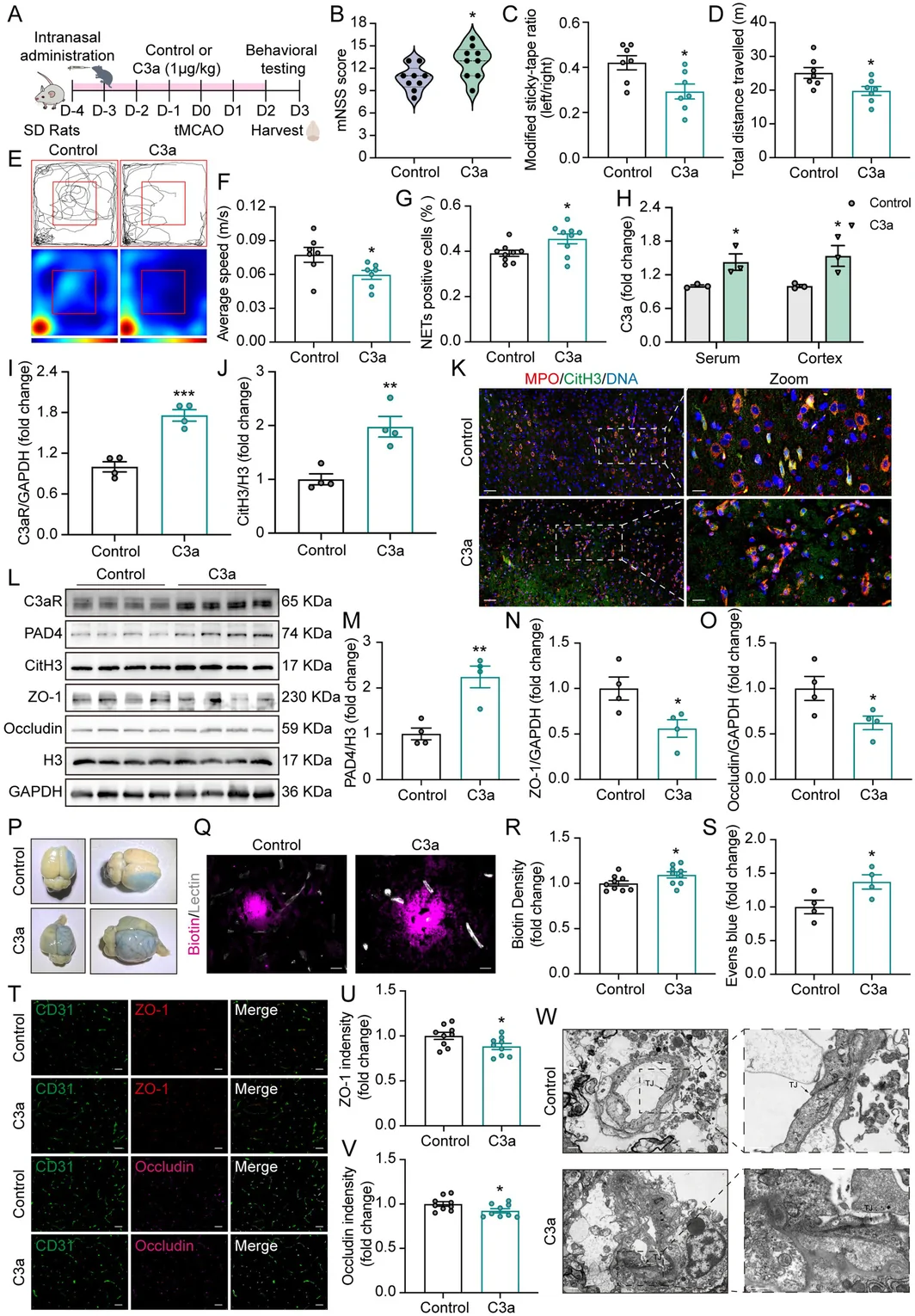
Activation of C3a‐C3aR signaling was associated with increased NETs formation, BBB damage, and neurological impairment after stroke. (A) The flow diagram of an experiment in this part. The animals received intranasal administration of C3a or vehicle for seven consecutive days, starting 4 days before tMCAO. On day 3 after tMCAO, behavioral tests were performed, and brain tissue was harvested. (B, C) mNSS score (*n* = 9) and MST ratio (*n* = 7) in the control and C3a groups. (D) Total distance in the control and C3a groups (*n* = 7). (E) Trajectory plots and open field movement heatmap in the control and C3a groups. (F) Average speed in the control and C3a groups (*n* = 7). (G) Quantification of NETs in the control and C3a groups (*n* = 9 FOVs of 3 rats, 3 FOVs per rat). (H) Levels of C3a in blood and cortex in the control and C3a groups (*n* = 3). (I, J) Quantification of C3aR and CitH3 expression in the control and C3a groups (*n* = 4). (K) Images of NETs in the control and C3a groups. Scale bar = 50 μm or 20 μm (zoom). (L) WB bands in the control and C3a groups. (M–O) Quantification of PAD4, ZO‐1, and Occludin expression in the control and C3a groups (*n* = 4). (P) Image of BBB leakage using Evans blue dye in the control and C3a groups. (Q) Image of BBB leakage using biotin staining in the control and C3a groups. Scale bar = 20 μm. (R, S) Quantification of biotin staining (*n* = 9 FOVs of 3 rats, 3 FOVs per rat) and Evans blue dye (*n* = 4) in the control and C3a groups. (T) Images of CD31 co‐labeled with ZO‐1 or Occludin in the control and C3a groups. Scale bar =20 μm. (U, V) Quantification of CD31 co‐labeled with ZO‐1 or Occludin in the control and C3a groups (*n* = 9 FOVs of 3 rats, 3 FOVs per rat). (W) Ultrastructure alteration of the tight junction near the endothelial cell. Scale bar = 1 μm or 0.5 μm (zoom). C3a group versus control group, data as mean ± SEM. *p < 0.05, **p < 0.01, and ***p < 0.001.

## 4 Discussion

This study provides evidence that C3a‐C3aR signaling is associated with NETs formation, BBB damage, and neurological injury after stroke. Specifically, stroke was associated with BBB damage, complement activation, increased C3a‐C3aR signaling, and increased NETs formation. Increased C3a‐C3aR signaling and NETs formation were associated with impaired BBB integrity following stroke. Inhibition of NETs formation with Cl‐amidine hydrochloride was associated with improved BBB dysfunction and neurological injury after stroke. Inhibition of C3a‐C3aR signaling with SB290157 was associated with reduced NETs formation, BBB dysfunction, and neurological injury in the experimental paradigm examined here. Intranasal C3a administration was associated with increased NETs formation, BBB disruption, and neurological damage in the experimental paradigm examined here. These findings support an association between C3a‐C3aR signaling, NETs formation, BBB disruption, and neurological injury after stroke.

Neutrophil extravasation across the damaged BBB plays an important role in disrupting BBB function [34]. In our study, after a stroke, we detected that BBB integrity was damaged, including BBB structure impairment and BBB leakage, accumulation of neutrophils in peripheral blood, and many neutrophils infiltrated into the brain parenchyma. Simultaneously, migrated neutrophils along the brain vessels released histones and proteases such as MPO and formed NETs. Notably, NETs have significant implications for BBB structure and integrity [34]. In brain disease conditions like traumatic brain injury (TBI) and sepsis‐associated encephalopathy (SAE), the formation of NETs is closely linked to elevated BBB permeability and subsequent inflammatory response [35, 36, 37]. Our work found that NETs formation is correlated with BBB disruption, decreased tight junction proteins, increased neuroinflammation, and poor neurobehavioral outcomes after stroke. When we employed Cl‐amidine hydrochloride to restrain NETs formation, these changes were all improved. Our findings show that NETs were increased after stroke and were associated with endothelial injury, reduced tight junction proteins, and BBB dysfunction; however, the present pharmacological data do not establish that NETs are the sole mediator of these effects. Other studies target suppressing NETs formation and offer BBB protection, promoting neuron survival, and inhibiting neuroinflammation in brain diseases including stroke and cerebral venous thrombosis [10, 38, 39]. Hence, NETs‐associated BBB disruption and neuroinflammation may contribute to stroke pathology. Targeting neutrophil recruitment or NETs formation or clearance may warrant further investigation as a strategy to mitigate BBB dysfunction during stroke.

RNA‐seq was employed in this study to conduct a transcriptome‐wide analysis of gene expression profiles in a stroke model. Several complement components were found to be significantly upregulated in the ischemic cortex by screening for DEGs. Additionally, KDA analysis discovered that C3aR was identified among the top 10 key drivers associated with the regulation of other genes, which draws our attention to the C3a‐C3aR axis. Moreover, in this study, C3 and C3aR were found to have significantly higher expression levels after stroke and be enriched in NETs formation and complement cascades, indicating a potential role for the C3a‐C3aR axis in the inflammatory response and NETs formation linked to stroke. C3 serves as the convergence point for all three complement activation pathways: classical, lectin, and alternative [40]. Upon activation, C3 is cleaved into C3a and C3b, both of which initiate various immune responses. Notably, C3a acts as a potent anaphylatoxin, recruiting and activating immune cells such as neutrophils [41], which can intensify inflammation. In the context of stroke, we also found the increased expression of C3 and C3aR identified in this study, as well as C5a, which indicates the whole complement was activated and neuroinflammatory cascades during stroke. ELISA and RT‐qPCR results show a great increase of complement and cytokines, which support a role for complement activation in the inflammatory response following stroke.

Meanwhile, we observed increased neutrophils migration into the brain parenchyma in association with increased C3a‐C3aR signaling. Firstly, we discovered that C3aR was present in the neutrophils. Secondly, after the stroke, numerous neutrophils were recruited into the brain through damaged BBB and formed NETs. Thirdly, Co‐IP results showed an association between C3‐derived material and C3aR in tissue lysates after stroke, without establishing direct ligand–receptor binding. Together, these findings support an association between C3a‐C3aR signaling, neutrophil responses, and NETs formation. Finally, we pharmacologically modulated C3a‐C3aR signaling and examined its effect on NETs formation and BBB disruption. Inhibition of C3a‐C3aR signaling by SB290157 was associated with reduced NETs formation and BBB damage. Additionally, intranasal C3a administration was associated with a reversal of the changes observed after SB290157 treatment. Thus, our findings support an association between C3a‐C3aR signaling, NETs formation, and BBB disruption. In an intracerebroventricularly lipopolysaccharide (LPS) induced local brain infection model, conventional C3 knock‐out and C3aR knock‐out mice showed that neutrophil migrating into the brain cortex and hippocampus was markedly decreased [42]. In other animal models of collagen antibody‐induced arthritis, C3aR knock‐out mice showed a lower percentage of synovial neutrophils [43]. Combined with this work, we deemed that C3a‐C3aR signaling is associated with neutrophil recruitment and NETs formation in inflammation‐related diseases and brain inflammation, such as stroke. These findings identify C3a‐C3aR signaling as a potential mechanistic target for further investigation. In the present peri‐ischemic paradigm, pharmacological modulation of C3a‐C3aR signaling was associated with changes in NETs formation, BBB integrity, and neurological outcomes. Importantly, the present study examined interventions initiated before or around ischemic injury and therefore does not establish efficacy when treatment is initiated after stroke onset. C3a‐C3aR signaling may also have phase‐dependent effects during stroke evolution; therefore, the findings here should be interpreted within the acute/peri‐ischemic context examined. Complement therapy has been proven to be valuable in preclinical models of some brain diseases, such as TBI, amyotrophic lateral sclerosis, and Alzheimer's disease [44, 45]. Current complement therapies in animal research, including complement inhibitors [46], neutralizing antibodies [44], targeted complement genes [47], and complement regulatory proteins, are powerful tools [48]. Accordingly, C3a‐C3aR signaling warrants further investigation as a potential target in stroke research, particularly using treatment paradigms initiated after stroke onset.

The current study has a number of limitations that should be noted. First, our pharmacological intervention tests do not prove that C3aR‐mediated BBB damage is particularly or totally dependent on neutrophil‐derived NETs, even though we do show a link between C3aR signaling, NETs production, and post‐stroke BBB impairment. Second, the current study did not employ neutrophil‐specific genetic modification of C3aR. As a result, it is impossible to totally rule out the possibility that C3aR signaling plays a role in other cell populations. Third, there were no conclusive mechanistic rescue studies that connected C3aR activation to NETs‐dependent BBB rupture, despite the fact that pharmacological inhibition of NETs synthesis reduced BBB damage. To further confirm the causal link between C3aR signaling, NETs formation, and BBB injury, more research employing neutrophil‐specific genetic methods and complementary rescue efforts will be necessary. Additionally, the effects of Cl‐amidine cannot be solely linked to PAD4‐dependent NETs formation because it is a pan‐PAD inhibitor rather than a highly selective PAD4 inhibitor. Furthermore, even though SB290157 was employed in this investigation as a C3aR antagonist, its pharmacological effects can vary depending on the situation. As a result, care should be taken while interpreting these pharmacological results.

## 5 Conclusion

In conclusion, this study indicates that C3a‐C3aR signaling is associated with NETs formation, BBB damage, and neurobehavioral impairment following stroke. Inhibition or activation of this pathway altered these outcomes in the peri‐ischemic experimental paradigms used here. Further studies using post‐stroke treatment, cell‐specific genetic manipulation, and mechanistic rescue approaches are needed to determine the causal and therapeutic significance of this pathway.

## Author Contributions

Qingtong Zhang: conceptualization, data curation, formal analysis, investigation, methodology, project administration, visualization, writing – original draft, writing – review and editing. Feng Xu: conceptualization, data curation, formal analysis, investigation, methodology, project administration, visualization, writing – original draft, writing – review and editing. Chenyao Jian: writing – review and editing, investigation, methodology, data curation. Nian Li: writing – review and editing, investigation, methodology, data curation. Tianhao Zhang: writing – review and editing, investigation, methodology. Daling Deng: writing – review and editing, investigation, methodology. Mingda Liu: writing – review and editing, investigation, methodology. Manlin Duan: writing – review and editing, supervision, project administration, conceptualization.

## Funding

This work was supported by the National Natural Science Foundation of China (Grant No. 82501886).

## Ethics Statement

All animal experimental protocols were granted by the Institutional Animal Care and Use Committee (IACUC) of Nanjing Medical University (Nanjing, China) with approval number IACUC‐2404060.

## Conflicts of Interest

The authors declare no conflicts of interest.

## Supporting information


**Figure S1:** RNA‐seq analysis of ischemic cortex and inflammatory response test on 3 days after stroke. (A) The flow diagram of an experiment in this part. tMCAO or sham rats were sacrificed to collect ischemic cortex for RNA‐seq analysis 3 days after inducing stroke (*n* = 3). We identified 474 DEGs, including 443 upregulated genes (93%) and 31 downregulated genes (7%). (B) Volcano map of DEGs (stroke group vs. sham group). Genes with |log_2_FC| > 1, *p*‐adjusted < 0.05, as DEGs, were highlighted in color. Those upregulated were shown on the right with red and downregulated genes are shown on the left with blue. (C–E) GO, KEGG, and Reactome enrichment bubble plots. All DEGs were included for GO (Biological processes), KEGG, and Reactome enrichment analysis. The Y‐axis shows enriched terms, and the X‐axis shows enrichment ratios. The bubble size indicates the gene number associated with terms, and the color gradient indicates the significance level. (F–J) GSEA analysis of GO or KEGG terms, including immune response, neutrophil chemotaxis, inflammatory response, NETs formation, and complement and coagulation cascades. GSEA analysis revealed that immune response, neutrophil chemotaxis, inflammatory response, NETs formation, complement, and coagulation cascades were significantly enriched, with a positive enrichment (NES > 0), indicating these are actively involved in the pathophysiology of stroke. (K) HE staining of brain slices from both groups. There are numerous neutrophils migrating into the brain parenchyma. Arrows represent the neutrophils with typical nuclear morphology. Scar bar = 50 μm or 20 μm (zoom). (L) Levels of inflammatory factors including TNFα, IL‐6, and IFN‐β in the ischemic cortex (*n* = 4 in the sham group and *n* = 5 in the stroke group). Stroke group versus sham group, data as Mean ± SEM. *p < 0.05 and ***p < 0.001.

**Table S1:** Primers of RT‐PCR.

## Data Availability

The data that support the findings of this study are available on request from the corresponding author. The data are not publicly available due to privacy or ethical restrictions.
