# Supplementary material for: C3a‐C3aR Signaling Modulates Neutrophil Extracellular Traps Formation and Contributes to Blood–Brain Barrier Injury After Stroke
Source: CNS Neurosci Ther. 2026 Nov 9;32(11):e71239. doi: 10.1002/cns.71239 (PMC13748817; doi:10.1002/cns.71239)
Supplement: Supplementary file 1 [file CNS-32-e71239-s001.docx]

Supplemental Figure 1. RNA-seq analysis of ischemic cortex and inflammatory response test on 3 days after stroke. (A): The flow diagram of an experiment in this part. tMCAO or sham rats were sacrificed to collect ischemic cortex for RNA-seq analysis 3 days after inducing stroke (n =3). We identified 474 DEGs, including 443 upregulated genes (93%) and 31 downregulated genes (7%). (B): Volcano map of DEGs (stroke group vs. sham group). Genes with |log_2_FC| > 1, *P*-adjusted < 0.05, as DEGs, were highlighted in color. Those upregulated were shown on the right with red and downregulated genes are shown on the left with blue. (C-E): GO, KEGG, and Reactome enrichment bubble plots. All DEGs were included for GO (Biological processes), KEGG, and Reactome enrichment analysis. The Y-axis shows enriched terms, and the X-axis shows enrichment ratios. The bubble size indicates the gene number associated with terms, and the color gradient indicates the significance level. (F-J): GSEA analysis of GO or KEGG terms, including immune response, neutrophil chemotaxis, inflammatory response, NETs formation, and complement and coagulation cascades. GSEA analysis revealed that immune response, neutrophil chemotaxis, inflammatory response, NETs formation, complement and coagulation cascades were significantly enriched, with a positive enrichment (NES > 0), indicating these are actively involved in the pathophysiology of stroke. (K): HE staining of brain slices from both groups. There are numerous neutrophils migrating into the brain parenchyma. Arrows represent the neutrophils with typical nuclear morphology. Scar bar = 50 μm or 20 μm (zoom). (L): Levels of inflammatory factors including TNFα, IL-6, and IFN-β in the ischemic cortex (n = 4 in the sham group and n = 5 in the stroke group). Stroke group vs. sham group, data as Mean ± SEM.**P* < 0.05 and ****P* < 0.001.
