# Supplementary material for: C3a‐C3aR Signaling Modulates Neutrophil Extracellular Traps Formation and Contributes to Blood–Brain Barrier Injury After Stroke
Source: CNS Neurosci Ther. 2026 Nov 9;32(11):e71239. doi: 10.1002/cns.71239 (PMC13748817; doi:10.1002/cns.71239)
Supplement: Supplementary file 2 [file CNS-32-e71239-s002.docx]

| Supplemental Table 1: Primers of RT-PCR | | |
| --- | --- | --- |
| RNA | Sequences forward/reversed 5’-3’ | |
| β-actin | Forward | CGTTGACATCCGTAAAGACCTC |
|  | Reversed | TAGGAGCCAGGGCAGTAATCT |
| C1qa | Forward | CAGGAATTCCCTTGGTTTCTG |
|  | Reversed | GATGCTGTCGGCTTCAGTACC |
| C3 | Forward | CGATGATCCTTGACATCTGCACC |
|  | Reversed | GTGGTGGTCAGTTGGGGCAGCCG |
| C3aR | Forward | TCATCCGGGAAATCATGGGC |
|  | Reversed | ATCTCCCCCTCTGTTCCATTC |
| C4 | Forward | AGACCCGCAACTTTCTGGTCC |
|  | Reversed | TGAGCTCAGGGATCCAGAGTC |
| C5 | Forward | GACCTGCAGCTCCTGCATCAGA |
|  | Reversed | CCTTCCCAACAGCATGCCTTTGT |
